# Boosting the Electrochemical Performance of Li- and Mn-Rich Cathodes by a Three-in-One Strategy

**DOI:** 10.1007/s40820-021-00725-0

**Published:** 2021-10-11

**Authors:** Wei He, Fangjun Ye, Jie Lin, Qian Wang, Qingshui Xie, Fei Pei, Chenying Zhang, Pengfei Liu, Xiuwan Li, Laisen Wang, Baihua Qu, Dong-Liang Peng

**Affiliations:** 1 State Key Lab of Physical Chemistry of Solid Surface, Collaborative Innovation Center of Chemistry for Energy Materials, College of Materials and Pen-Tung Sah Institute of Micro-Nano Science and Technology, Xiamen University, Xiamen, 361005 People’s Republic of China; 2 College of Chemistry and Chemical Engineering, Xiamen University, Xiamen, 361005 People’s Republic of China; 3 Zhengzhou Key Laboratory of Big Data Analysis and Application, Henan Academy of Big Data, Zhengzhou University, Zhengzhou, 450002 People’s Republic of China; 4 Fujian Provincial Key Laboratory of Light Propagation and Transformation, College of Information Science and Engineering, Huaqiao University, Xiamen, 361021 People’s Republic of China; 5 Shenzhen Research Institute of Xiamen University, Shenzhen, 518000 People’s Republic of China

**Keywords:** Li- and Mn-rich cathodes, Cation–polyanion co-doping, Defect and stress engineering, Good structure stability, Electrochemical performance

## Abstract

There are plenty of issues need to be solved before the practical application of Li- and Mn-rich cathodes, including the detrimental voltage decay and mediocre rate capability, etc. Element doping can effectively solve the above problems, but cause the loss of capacity. The introduction of appropriate defects can compensate the capacity loss; however, it will lead to structural mismatch and stress accumulation. Herein, a three-in-one method that combines cation–polyanion co-doping, defect construction, and stress engineering is proposed. The co-doped Na^+^/SO_4_^2−^ can stabilize the layer framework and enhance the capacity and voltage stability. The induced defects would activate more reaction sites and promote the electrochemical performance. Meanwhile, the unique alternately distributed defect bands and crystal bands structure can alleviate the stress accumulation caused by changes of cell parameters upon cycling. Consequently, the modified sample retains a capacity of 273 mAh g^−1^ with a high-capacity retention of 94.1% after 100 cycles at 0.2 C, and 152 mAh g^−1^ after 1000 cycles at 2 C, the corresponding voltage attenuation is less than 0.907 mV per cycle.

**Supplementary Information:**

The online version contains supplementary material available at 10.1007/s40820-021-00725-0.

## Highlights

- A novel three-in-one method is put forward to prepare Li- and Mn-rich cathode.
- The stress evolution of layered materials during cycling is characterized.
- The capacity and voltage stability are enhanced greatly.

**Supplementary Information:**

The online version contains supplementary material available at 10.1007/s40820-021-00725-0.

## Graphical abstract

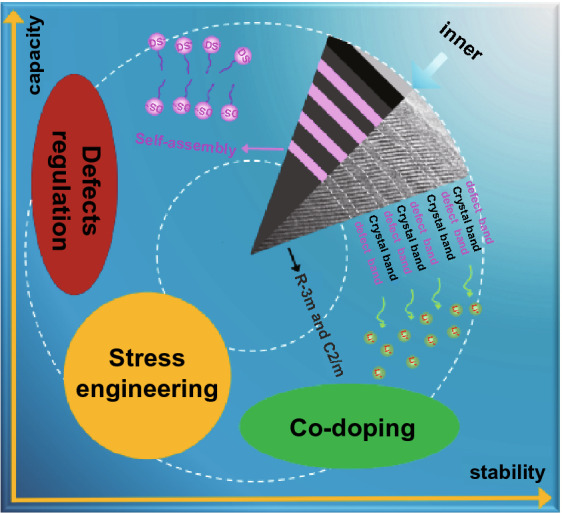

## Introduction

With the rise of electric devices, electric vehicles and large grid energy storage, etc., the lithium-ion batteries (LIBs) industry is developing vigorously [1–5]. However, the development of cathode materials is far below the market expectations, and it is increasingly unable to meet the energy storage demands [6–8]. Due to its high theoretical specific capacity (generally over 300 mAh g^−1^), environmental friendly, low cost and other advantages, the Li- and Mn-rich cathodes (LMR) with the chemical formula of Li_1+*x*_(Ni, Mn, Co)_1−*x*_O_2_ have received a lot of attention, and been regarded as one of the most promising cathode material for future LIBs [9–12]. However, LMR cathodes suffer from severe capacity/voltage fading, poor rate capability, and low initial Coulombic efficiency [1].

Tremendous efforts have been applied to solve above-mentioned problems, such as doping by other transition metal (TM) elements or polyanions, which is effective to obtain excellent cyclability and suppress the abominable voltage fading [13–15]. Nevertheless, the incorporated doping of inactive elements usually reduces the reversible capacity. On the other hand, some previous works have implied that introducing defects into the LMR cathode materials, like the planar defects of stacking faults, can accommodate strain and stress in the layered crystal grains [16–20]. These defects in the LMR cathodes have been confirmed as active sites during charge and discharge processes, which means that the introduction of appropriate defects in LMR cathode can compensate the capacity loss caused by elemental doping [15]. However, these modification methods have little effect on eliminating the damage caused by stress accumulation during cycling.

Herein, a three-in-one method by combining cation–polyanion co-doping, defect, and stress engineering is put forward to solve these issues. Defective structures are pre-constructed in LMR materials by the self-assembly behavior of sodium dodecyl sulfate (SDS, an anionic surfactant) and the subsequent high-temperature calcination process. The SDS will decompose and endow cation–polyanion (Na^+^/SO_4_^2−^) co-doping into the LMR materials to stabilize the layered structure, which can improve the structure stability and lifespan of the LMR cathode greatly. Finally, a state where surface defect bands and crystal bands are alternately distributed will form. This new structure can not only change the traditional 2D long ion diffusion path of layered LMRs, increase the diffusion outlet, but also delay and release the stress accumulation during cycling. As a result, the discharge capacities of the modified sample (D-LMR) reach to 273 mAh g^−1^ with a capacity retention of 94.1% after 100 cycles at 0.2 C and 152 mAh g^−1^ with a high-capacity retention of 79.6% after 1000 cycles even at 2 C. The voltage fading has also been dramatically suppressed, and the voltage retention is as high as 74.5% after 1000 cycles with a very low attenuation rate of 0.907 mV per cycle. Undoubtedly, compared to the pristine sample (P-LMR), the capacity and voltage stabilities of the modified D-LMR sample are enhanced significantly. These results above demonstrate the effectiveness of this three-in-one method in inhibiting voltage decaying, enhancing ion diffusion, and then improving the comprehensive electrochemical properties of LMR cathodes.

## Experimental Section

### Materials Synthesis

#### Synthesis of P-LMR Sample

The P-LMR cathode material (Li_1.2_Mn_0.54_Ni_0.13_Co_0.13_O_2_) is prepared by a typical co-precipitation method combing with the subsequent high-temperature calcination process. All chemicals are purchased from Macklin Biochemical Co., Ltd. (Shanghai, China), which are analytically pure and without further treatment. According to the stoichiometric ratio (2/2/4 mmol), the chlorate of nickel, cobalt, and manganese were dissolved in 120 mL of deionized water and ultrasound treated for 5 min, then, kept stirring until completely dissolved. The solution above was denoted as solution A. 60 mL of ammonium carbonate (5% excess) solution as precipitant is denoted as solution B. Solution B was added into solution A drop by drop and kept stirring for 12 h at room temperature. The slurry was centrifugally cleaned with deionized water and anhydrous ethanol for 3 to 5 times to collect the solid precipitation and dried in an oven at 80 °C for 12 h to obtain the carbonate precursor (denoted as P-NCMCO). The P-NCMCO was pre-heated at 500 °C for 5 h with a heating rate of 2 °C min^−1^ to obtain oxide precursor denoted as P-NCMO. Finally, the P-NCMO was mixed with an appropriate amount of lithium carbonate (3% excess) and ground evenly, calcined at 800 ℃ for 12 h to obtain the product of P-LMR.

#### Synthesis of D-LMR Sample

The schematic diagram of the preparation process for D-LMR is shown in Figs. S1 and S2. Firstly, 30 mL of the ammonium carbonate solution was dropped into TM ions solution via valve A (Fig. S2) and kept stirring for 5 min to form large numbers of carbonate precursor particles which would act as hydrophobic centers. Secondly, 1 g of sodium dodecyl sulfate (SDS) powder was dissolved into 30 mL deionized water and the obtained anionic surfactants solution of SDS was added into the above solution via valve B (Fig. S2). Then, the rest 30 mL of the ammonium carbonate solution was added into above solution drop by drop and kept stirring for 12 h, so that TM ions would precipitate and nucleate on the surface of DS^−^. Thirdly, the supernatant of the carbonate precursor was sucked out after the standing and layering for a while. The subsequent calcination process is the same as the synthesis of P-LMR.

### Materials Characterization

The crystal structure analysis of all samples was investigated by X-ray diffraction (XRD, Ultima IV-185, Rigaku, Japan) with Cu Kα-radiation (λ = 1.5406 Å), at 25 mA and 40 kV. The Rietveld refinement analysis was carried out by the EXPGUI-GSAS program [21, 22], the data were detected at a scan speed of 1° min^−1^ between the 2-theta range of 10°–100°. The thermal stability of the materials was tested by the synchronous thermal analyzer (DSC/DTA-TG STA 449 F5, NETZSCH, Germany). The chemical valence analyses were detected by X-ray photoelectron spectroscopy (XPS, PHI Quantum 2000). The Fourier transform infrared spectroscopy (FTIR, Nicoletis10) and the Raman spectrum (Xplora, the wavelength is 638 nm) of cathode materials were used to identify the micro-zone structure. The arrangement of atomic lattice and the distribution of elements were tested by the field-emission scanning electron microscopy (FESEM, SUPRA-55, ZEISS, Germany) and high-resolution Tecnai F30 field transmission electron microscopy (HRTEM, TECNAI-F30, Philips-FEI, Netherlands) at a working voltage of 300 kV.

### Electrochemical Test

The cathode electrode was made up of 80% active cathode materials, 10% acetylene black as conductive agent and 10% polyvinylidene fluoride (PVDF) as binder, which was coated on an carbon-coated-Al current collector. Then, the Al foil collector was punched into the disks with a diameter of 12 mm and assembled with Li metal plates in an Ar-filled MBraun glovebox (MB-10-G-V2A, H_2_O < 0.5 ppm, O_2_ < 0.5 ppm). The Celgard 2500 polypropylene was used as separator and a commercial electrolyte of 1 M LiPF_6_ dissolved in a mixture of EC and DEC with a volume ratio of 1:1 with 5% FEC addition was used as electrolyte. The loading density of LMR cathode was about 1.5 mg cm^−2^ and its electrochemical properties are tested by the NEWARE battery cycler (CT-4800 T-5V10mA-164, Shenzhen, China) between 2 and 4.8 V at room temperature (30 °C). The electrochemical impedance spectroscopy (EIS) measurements were measured using an electrochemical workstation (CHI760E, Shanghai Chenhua Instrument Corp., China) with a frequency from 100 kHz to 0.01 Hz.

## Results and Discussion

**Fig. 1:**
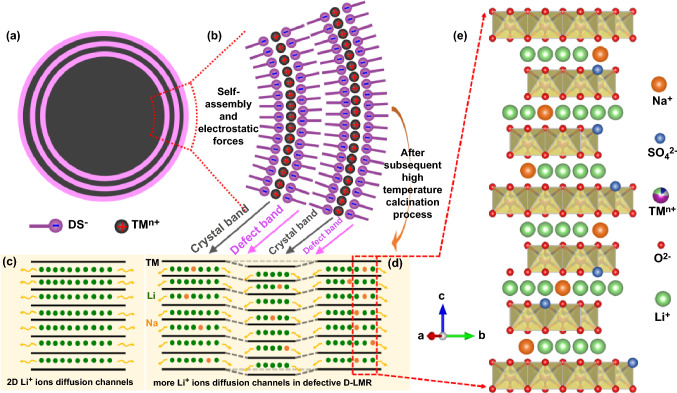
Schematic diagram of the three-in-one design strategy. **a** Schematic diagram of 2D cross-sectional view for the surface structure of carbonate precursor (denoted as D-NCMCO hereafter). **b** On-site generation of the TM^2+^(DS^−^)_2_ vesicles on the surface of TM^2+^(CO_3_^2−^) nano-grains to form the TM^2+^(CO_3_^2−^)_*x*_(DS^−^)_*y*_ colloidosomes. **c** Schematic depictions of the conventional long 2D Li^+^ ions diffusion channels in P-LMR and **d** the more channels in D-LMR samples with alternate defect band. **e** Sites of the doped elements in the unit cell of LMR crystal

The design blueprint for this three-in-one strategy is shown in Fig. 1. The firstly nucleated grains of carbonate precursor act as hydrophobic cores, the amphiphilic DS^−^ groups will be centered around, the hydrophobic end faces inward, and the hydrophilic end faces outward, showing radial assembly [23–26]. See Note S1 in Supporting Information for more details about the mechanism of SDS self-assembly behaviors. With the addition of precipitant, the final distribution state is formed, as shown in Fig. 1a, b. The thermodynamic properties of SDS are studied in detail and shown in Fig. S3, in which the decomposition temperature of SDS is about 257.4 °C, and the final product is Na_2_SO_4_. So that, the cation–polyanion (Na^+^/SO_4_^2−^) co-doping is introduced into the LMR cathode after the subsequent high-temperature calcination process. The schematic diagram of the conventional long 2D Li^+^ ions diffusion channels in P-LMR is shown in Fig. 1c. Compared with this kind of direct channel structure, after the three-in-one method modification, a defect band and crystal band alternately arranged structure is constructed in the outer surface of grains, which can slow-release the accumulated interior stress, and build numerous side ion diffusion channels (Fig. 1d). The distribution of the various ions in the final bulk phase of the D-LMR material is shown in Fig. 1e. Combined with cation–polyanion doping, defect construction, and the stress regulation, the electrochemical properties of the LMR cathode have been improved greatly.

**Fig. 2:**
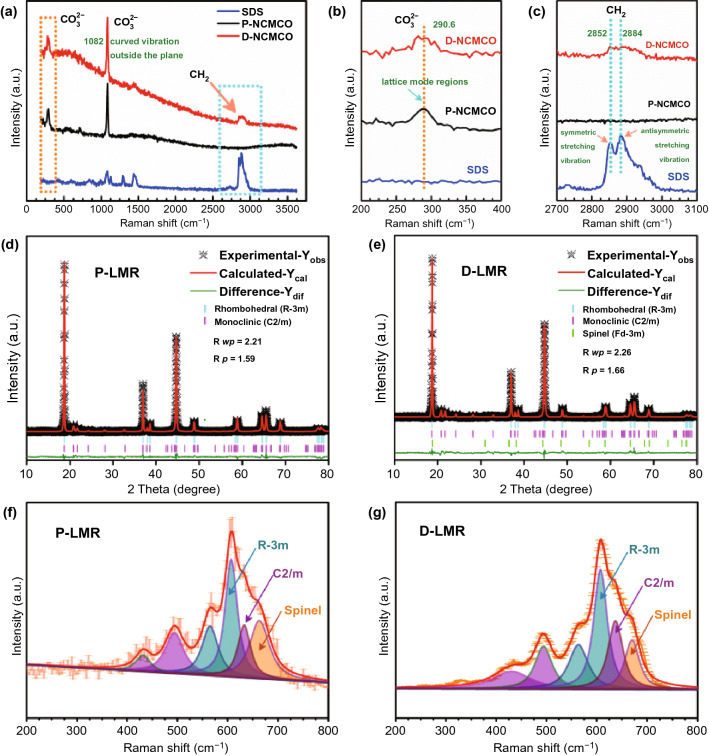
Various spectral characterization. **a**–**c** Raman spectra of P-NCMCO, D-NCMCO, and pure SDS powders. The Rietveld refinement results of **d** P-LMR and **e** D-LMR samples. The fitted results of Raman spectra for **f** P-LMR and **g** D-LMR

The Raman spectra of carbonate precursors for pristine (P-NCMCO), modified (D-NCMCO) LMR samples, and pure SDS powders (Fig. 2a) are used to confirm the presence of surfactants in the precursors. The peak at 290.6 cm^−1^ (Fig. 2b) can be indexed to the characteristic vibration of CO_3_^2−^, which exists only in carbonate precursors for P-NCMCO and D-NCMCO, but not in SDS. The two split peaks at 2852 and 2884 cm^−1^ (Fig. 2c) that only exist in pure SDS and D-NCMCO samples are, respectively, indexed to the symmetric stretching vibration and antisymmetric stretching vibration of -CH_2_- group, proving that SDS does exist in D-NCMCO. The powder XRD patterns of carbonate precursors and the corresponding oxide precursors are shown in Fig. S4, which match well with their standard PDF card (NiCO_3_ PDF#12-0771, MnCO_3_ PDF#44-1472, CoCO_3_ PDF#11-0692). The corresponding Rietveld refinement results are illustrated in Fig. 2d, e. The obtained cell parameters shown in Table S1 demonstrate that both the *c*-axis spacing distance and the cell volume of D-LMR increase, which is due to the co-doping of cations and polyanions. The fitted results of Raman spectra for P-LMR and D-LMR are shown in Fig. 2f, g. The spinel-like component in P-LMR has a larger proportion compared with D-LMR, indicating an effective enhancement in inhibiting the phase transformation. The SEM images of the carbonate and oxide precursors are shown in Fig. S5, the precursors of both two samples have similar morphology and wide particle size distribution. The products present a hierarchical spherical structure, Na and S elements can be clearly detected in the elementary mappings (Fig. S6) and the EDS curves (Fig. S7). The contents of Mn/Co/Ni in P-LMR and D-LMR basically conform to the stoichiometric ratios (Table S2). The Fourier transform infrared (FTIR) spectroscopy results for P-LMR and D-LMR (Fig. S8) also confirm the existence of SO_4_^2−^ in D-LMR.

**Fig. 3:**
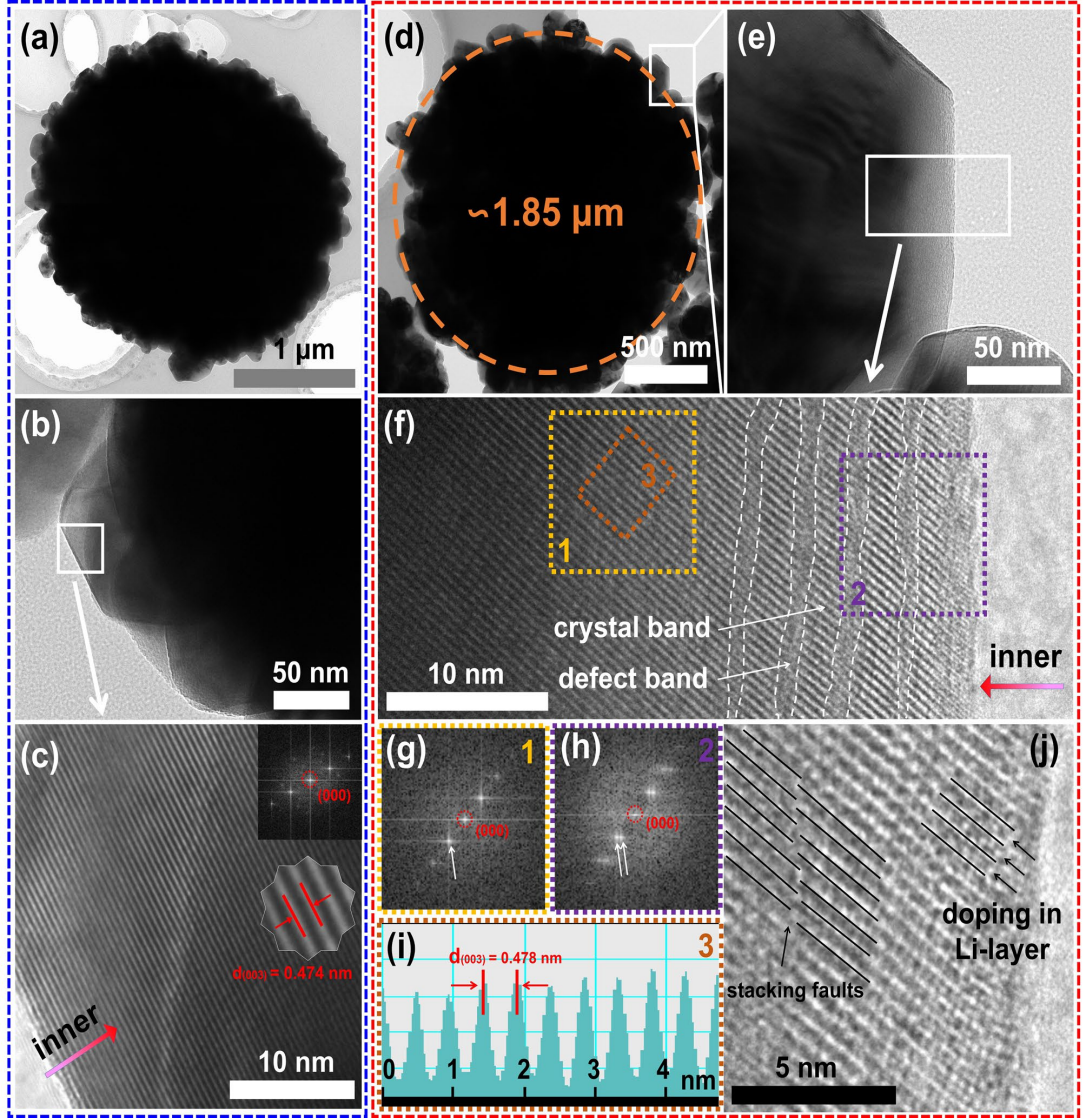
HRTEM characterization of the products. **a** TEM images of the secondary particle, **b** primary particles, and **c** HRTEM images for P-LMR samples. **d** TEM images of the secondary particles, **e** primary particles, and **f** HRTEM images for D-LMR samples. **g**, **h** Corresponding FFT modes for region 1 and region 2 in **f**. **i** Corresponding lattice space for region 3 in **f**. **j** Enlarged demonstration of defect bands for D-LMR sample

The TEM images of P-LMR are shown in Fig. 3a, b. Figure 3c illustrates the lattice fringes from the surface to the interior of the bulk phase for P-LMR, showing the perfect layered structure. The lattice spacing of 0.474 nm is exactly indexed to the (003) plane. The HRTEM images of D-LMR are displayed in Fig. 3d, e. Both P-LMR and D-LMR have similar morphology. However, the lattice fringes from the surface to the near-surface of the primary gains in D-LMR demonstrate an alternate distribution of defect bands and crystal bands (Fig. 3f). The inner core remains an intact layered structure. This phenomenon is also confirmed by the corresponding diffraction spots. The typical diffraction spots pattern of hexagonal notation in Fig. 3g for region 1, and the split weak spots localized near the diffusion spots pattern of hexagonal notation in Fig. 3h for region 2 are clearly seen. Note that the split diffusion spots in the electron diffraction patterns could be the signatures of stacking faults in the ordered layer-structures occurred along the *c*-axis of monoclinic phase. The HRTEM image of region 3 (Fig. 3i) shows an expanded lattice spacing distance of (003) facets, indicating the lattice doping of Na^+^. The enlarged view of region 2 (Fig. 3j) shows that the lattice streaks of (003) plane is misaligned on both sides of the defect band, that is stacking faults, which is consistent with the results of electron diffraction spots.

**Fig. 4:**
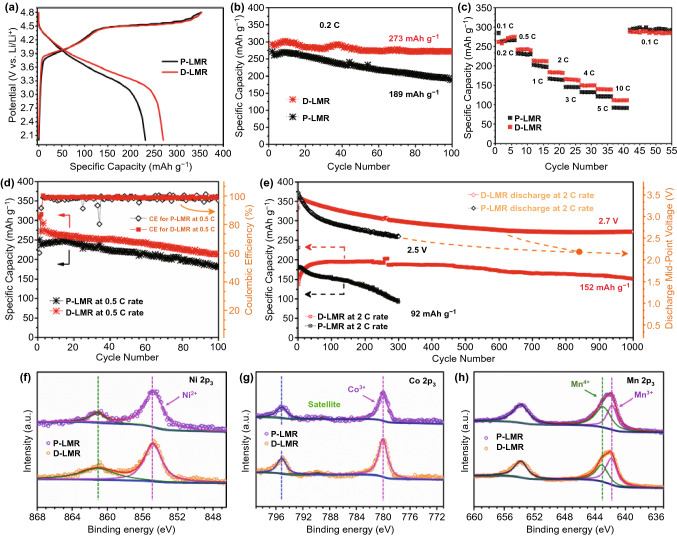
Electrochemical performance data. **a** Charge–discharge curves for the first cycle of P-/D-LMR at 0.1C. **b** Cycling performance at 0.2 C, **c** rate performance and **d** long-term cycling performance at 0.5 C for P-LMR and D-LMR samples. **e** Cycling stability and corresponding discharge mid-point voltages for P-LMR and D-LMR electrodes at 2 C rate. The XPS spectra of P-LMR and D-LMR cathode materials of **f** Ni 2p_3_, **g** Co 2p_3_, and **h** Mn 2p_3_

The initial charge/discharge curves of both two samples at 0.1 C (Fig. 4a) exhibit typical characteristics of LRM-layered oxides, a slope and long plateau at 4.5 V [27]. The D-LMR cathode delivers high discharge capacity of 273 mAh g^−1^ with a superior capacity retention of 94.1% after 100 cycles at 0.2 C, which is much better than that of P-LMR (189 mAh g^−1^ with a capacity retention of 69.5%). The similar results also appear at 0.5 C (Fig. 4d). Furthermore, the modified D-LMR electrode shows better rate performance than P-LMR as shown in Fig. 4c. Compared with P-LMR electrode, the D-LMR cathode provides a capacity of 152 mAh g^−1^ with a high-capacity retention of 79.6% after 1000 cycles at a high rate of 2 C. What’s more, the voltage fading has also been dramatically suppressed. The voltage retention is as high as 74.5% after 1000 cycles at 2 C rate and the attenuation is less than 0.907 mV per cycle for D-LMR cathode (Fig. 4e). These results suggest that this three-in-one method plays a positive role in improving the comprehensive electrochemical properties of D-LMR cathode materials.

The electrochemical impedance spectra (EIS) of P-LMR and D-LMR electrodes before cycling and after 10 cycles are measured (Fig. S9). The diffusion coefficients of Li^+^ (*D*_*Li*_^+^) and the fitted impedance results for P-LMR and D-LMR electrodes are summarized in Table S3 (see Note S2 in Supporting Information for detailed calculation process). The results show that the ionic diffusion coefficient of D-LMR increases from 1.99** × **10^–15^ to 7.00** × **10^–15^ cm^2^ s^−1^ after 10 cycles, which is larger than that of P-LMR (from 4.64** × **10^–17^ to 1.74** × **10^–16^ cm^2^ s^−1^). In addition, the charge transfer impedance (R_ct_) of P-LMR changes a lot while the D-LMR keeps stable at a lower level after cycling. The stable and fast ion diffusion rate and the lower impedance for D-LMR would be owing to the novel modification method. The pre-constructed surface defect bands can provide more lateral diffusion channels during the insertion/extraction processes, which is equivalent to increase the active reaction sites. The doped inert Na^+^ will occupy the Li sites and stay in the Li-layer to support the layer structure after Li^+^ extraction, and the doped SO_4_^2−^ will occupy the sites of lattice oxygen, which can inhibit the irreversible release of the lattice oxygen during cycling, enhance the bonding ability with TM ions, and prevent the migration of TM ions to Li-layers effectively, finally suppressing the occurrence of the phase transition from layer to spinel and finally to rock-salt phase.

The valence state of various elements in LMR materials before cycling are analyzed by XPS. The full-spectra of P-LMR and D-LMR are shown in Fig. S10a. The characteristic peaks of Na 1s and S 2p are observed clearly in D-LMR sample (Fig. S10b, c), proving the co-doping in the product. The peaks at 529.24 and 531.50 eV for O 1s (Fig. S10d) can be attributed to the binding effect of O-TMs in the lattice and oxygen on the surface of the material, respectively. The peaks at 284.61, 285.25, and 288.62 eV for C 1s (Fig. S10e) can be recognized as C–C, C–OH and C=O, respectively [28, 29]. The peak intensity of D-LMR at 531.50 eV (O–Li) and 288.62 eV (C=O) is lower than that of P-LMR, which is due to the replacement of surface oxygen by polyanion (SO_4_^2−^) and the decreased detrimental residual lithium (Li_2_CO_3_) on the surface. Additionally, the Ni 2p_3_, Co 2p_3_, and Mn 2p_3_ spectra are shown in Fig. 4f–h. The peaks at 854.86 and 780.01 eV are assigned to 2p_3_ of Ni^2+^ and Co^3+^. The binding energies of Ni^2+^ and Co^3+^ in P-LMR and D-LMR have no significant difference. The divided peaks at 641.75 and 643.12 eV of Mn 2p spectrum in Fig. 4h correspond to the Mn^3+^ and Mn^4+^, respectively. Clearly, the peak intensity of Mn^3+^ for D-LMR is higher than P-LMR, which is resulted from the polyanion doping, the valence of Mn^4+^ is reduced to maintain the charge balance [30, 31]. And this behavior would also activate some of the lattice oxygen, which is one of the origins for the high specific capacity of D-LMR.

**Fig. 5:**
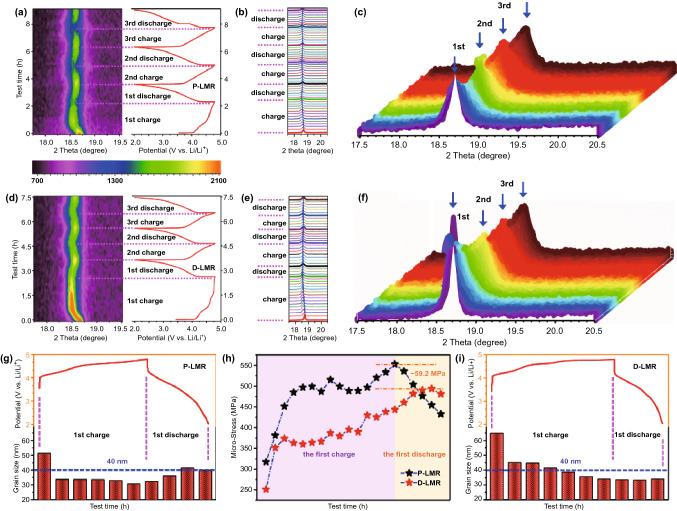
In-situ XRD spectra and 2D contour plot of the first three cycles for **a** P-LMR and **d** D-LMR. The corresponding magnified details of (003) peak for **b**, **c** P-LMR and **e**, **f** D-LMR during cycling. The change of grain size at different SOC during the first cycle for **g** P-LMR and **i** D-LMR. **h** Corresponding micro-stress images of the P-LMR and D-LMR samples during first charge and discharge cycle, calculated from the in-situ XRD results

Figure 5a, b, d, e demonstrates the in-situ XRD patterns (color-coded contour map) and corresponding plots during the first three cycles for P-LMR and D-LMR, respectively. The spacing distance change along *c*-axis (elongation and shrinkage) is reflected by the shift of (003) peak, which also suggests the underlying phase transformation during cycling. The shift of (003) peak to lower angle from the open-circuit voltage to 4.5 V corresponds to the increase of interlayer spacing (elongation), which can be contributed to the enhancement of the electrostatic repulsive force between the O-layers due to the extraction of Li^+^ ions from the Li-layers, resulting in the unit cell expansion along the c-axis [13]. When charging from 4.5 to 4.8 V, the (003) peak shifts to high angle, which corresponds to the decrease of interlayer spacing (shrinkage). This variation of (003) peak relates to the release of oxygen and the extraction of Li^+^ ions from the TM-layers during the activation process of monoclinic phase (Li_2_MnO_3_). From the 3D color-coded contour maps (Fig. 5c, f), the swing of (003) peak for D-LMR sample is slighter than that of P-LMR sample, indicating the better stability of layered framework after modification, which can promote the capacity and voltage stability as shown in Fig. 4.

The respiration of lattice constants during cycling that confirmed by in-situ XRD results above will induce the interior stress in the LRM material, which would be harmful to the structural stability (cause cracks) and result in deterioration of cycling performance. However, the improved electrochemical performance of D-LMR electrode confirms that this three-in-one method will help in stabilizing the capacity/voltage and accelerating the Li^+^ diffusion kinetics during long-term cycles. The reason is that this method can regulate the accumulation of strain, which would eliminate the deterioration to the material properties caused by the internal strain during cycling. In order to quantitatively elucidate the mechanism of stress engineering in helping to relieve the structure deterioration of D-LMR, the nonlinear dynamic variation of the internal strains from the first cycle of *in-situ* XRD is analyzed (see Note S3 in Supporting Information for more details about the calculation process of the corresponding stress) [32, 33]. For P-LMR sample, during the charge process, the interior stress (Fig. 5h) increases gradually and reaches to summit at the cutoff voltage. Inversely, the stress decreases continuously during the subsequent discharge process. However, the tendency of stress variation in D-LMR materials is quite different. The stress does not reach its maximum at the end of charge process, but still increases at the beginning of discharge process, indicating that the stress accumulation is slowed down and the arrival of the stress peak value is delayed in the modified D-LMR with alternative distribution of crystal and defect bands. Moreover, the internal micro-stress in D-LMR is all less than P-LMR during cycling, the peak value of D-LMR is 59.2 MPa lower than P-LMR sample. Similar results are also found on time–strain curves (Fig. S11) and time–dislocation density curves (Fig. S12).

Additionally, according to the data of in-situ XRD, the size change of single-crystal particles along the *c*-axis at different state of charge (SOC) during the first cycle is also compared quantitatively. For P-LMR sample (Fig. 5g), with the removal of Li^+^ from electrode material, the grain size decreases gradually and reaches the minimum value at the end of charge process. After that, the particle size increases gradually with the discharge process going on. However, a different situation occurs in D-LMR sample (Fig. 5i). During charge process, the grain size also decreases gradually and reaches the minimum value at the end of charging. Noticeably, during the discharge process of D-LMR, the grain size does not increase continuously like P-LMR sample, but maintains a relatively stable size distribution. This is due to the relieved interior stress by the novel alternately distributed crystal and defect band structure, and the enhancement effect of cation/polyanion co-doping on the stabilization of the layer framework, which effectively alleviates the deterioration of the material property caused by lattice shrinkage and expansion.

## Conclusions

In this work, we develop a simple and effective three-in-one method to synthesize Li-rich cathode materials via the self-assembly behavior of SDS and the subsequent high-temperature calcination process. The decomposition of SDS will lead to the cation/polyanion co-doping to enhance the structural stability and improve the Li^+^ conductivity. By pre-constructing alternately distributed defect bands and crystal band structure to change the traditional 2D ion diffusion model of long channel path, and increasing lots of longitudinal diffusion outlet, the rebuilt 3D ion diffusion model can promote the Li^+^ diffusion significantly. Meanwhile, this special structural design can also delay and relieve the accumulation of stress caused by the volume change during long-term cycling. By combining the advantages of cation/polyanion co-doping, defect, and stress engineering, the lifespan and the voltage stability of the D-LMR cathode material are dramatically promoted. A large reversible capacity of 152 mAh g^−1^ with a high-capacity retention of 79.6% is obtained even after 1000 cycles at 2 C, while the voltage fading is less than 0.907 mV per cycle. Above all, the anionic surfactant that used in this three-in-one method is very common and low cost, delivering a possible roadmap for the development of high-capacity LMR cathode materials for next-generation LIBs.

## Supplementary Information

Below is the link to the electronic supplementary material.

Supplementary file1 (PDF 1433 kb)
