# Supplementary material for: Boosting the Electrochemical Performance of Li- and Mn-Rich Cathodes by a Three-in-One Strategy
Source: Nanomicro Lett. 2021 Oct 11;13:205. doi: 10.1007/s40820-021-00725-0 (PMC8505566; doi:10.1007/s40820-021-00725-0)
Supplement: Supplementary file 1 — Supplementary file1 (PDF 1433 kb) [file 40820_2021_725_MOESM1_ESM.pdf]

Supporting Information for

Boosting the Electrochemical Performance of Li- and Mn-Rich Cathodes by A Three-in-One Strategy

Wei He[1], Fangjun Ye[1], Jie Lin[1], Qian Wang[1], Qingshui Xie[1, 5, *], Fei Pei[2], Chenying Zhang[1], Pengfei Liu[3], Xiuwan Li[4], Laisen Wang[1, *], Baihua Qu[1, 5, *], Dong-Liang Peng[1]

[1]State Key Lab of Physical Chemistry of Solid Surface, Collaborative Innovation Center of Chemistry for Energy Materials, College of Materials and Pen-Tung Sah Institute of Micro-Nano Science and Technology, Xiamen University, Xiamen 361005, P. R. China

[2]College of Chemistry and Chemical Engineering, Xiamen University, Xiamen, 361005, P. R. China

[3]Zhengzhou Key Laboratory of Big Data Analysis and Application, Henan Academy of Big Data, Zhengzhou University, Zhengzhou 450002, P. R. China

[4]Fujian Provincial Key Laboratory of Light Propagation and Transformation, College of Information Science and Engineering, Huaqiao University, Xiamen 361021, P. R. China

[5]Shenzhen Research Institute of Xiamen University, Shenzhen, 518000, P. R. China

*Corresponding authors. E-mail: xieqsh@xmu.edu.cn (Q. Xie), wangls@xmu.edu.cn (L. Wang), bhqu@xmu.edu.cn (B. Qu)

Note S1 Mechanism of SDS Self-assembly Behavior

In the water solution, the SDS molecules will separate into negatively charged DS^- units (CH_3-(-CH_2-)$_{11}$-O-SO_3^-) and the positively charged Na^+. As is known to all, the water-soluble ions are always hydrophilic, whereas organic groups are usually hydrophobic. The DS^- units contain hydrophobic end (long-chain alkyl, CH_3-(-CH_2-)$_{11}$-) and hydrophilic end (sulfonate, -O-SO_3^-), and are radially distributed where the hydrophilic moiety facing outward to directly contact with the water and the hydrophobic moiety facing inward to stay away from the water. By regulating the concentration and temperature, the DS^- ions will spontaneously form micelles or vesicles. Herein, the hydrophobic end of the DS^- ions (the long chain alkyl, CH_3-(-CH_2-)$_{11}$-) will adsorb on the particle surface of the carbonate precursor, while the hydrophilic end (sulphuric acid radical, -O-SO_3^-) is radially distributed on the particle surface toward the water solvent. TM^{n+} ions (positive charged) in the solution will be adsorbed on the surface of DS^- ions (negative charged). By adjusting the concentration of SDS, the morphology of the carbonate precursor and DS ion self-assembly layer alternative distribution is formed.

Note S2 Calculation of Ionic Diffusion Coefficient

The diffusion coefficient of lithium ions (D_{Li^+}) represents the diffusion ability of Li^+, which could be calculated according to the following Equation:

$$D_{Li^+} = \frac{R^2T^2}{2c^2F^4A^2\sigma^2} \quad \text{(S1)}$$

wherein R is the gas constant (8.314 J mol^{-1} K^{-1}), T is the absolute temperature (here is 301.15 K), c is the concentration of Li^+ in the material (for this cathode it is 4.96×10^{-2} mol cm^{-3}), F is the Faraday constant (96485.34 C mol^{-1}); A is the surface area of the electrode (for this electrode it is 1.131 cm^2), and σ is the Warburg factor follow the nether relationship:

$$Z' = R_{ct} + R_s + \sigma\omega^{-1/2} \quad \text{(S2)}$$

Z' is the real part of impedance, thus σ can be obtained from the linear fitting of Z' vs. $\omega^{-1/2}$ in the low frequency range shown in Fig. S8 [S1, S2].

Note S3 Nonlinear dynamic variation of the interior strains/stress were calculated from the results of *in-situ* XRD by Origin 8.5 through the following Equation:

$$\sigma = E\cdot\varepsilon \quad \text{(S3)}$$

wherein E is the elastic coefficient (Young's modulus) of the LMR material, after referring to relevant literature, the value here is 76312 MPa, the σ and ε values stand for the corresponding stress and strain, respectively [S3]. Thus, to obtained stress, the values of strain must be got. Firstly, the (003) peak positions (2θ, Table S4) of the corresponding *in-situ* XRD curves were collected by Origin, as well as the full width at half maxima (FWHM, β, Table S5). Then, the corresponding particle size (D, nm) that along (003) direction can be obtained by the following Scherer Equation:

$$D = \frac{k\lambda}{\beta Cos\theta} \quad \text{(S4)}$$

wherein the λ is the Cu Kα-radiation (λ= 0.15406 nm), k is a constant (here is 0.9). The dislocation density δ ($\times10^{-3}$, nm^{-2}) and micro strain ε ($\times 10^{-3}$) are calculated by the following Equation:

$$\delta = \frac{1}{D^2} \quad \text{(S5)}$$

$$\varepsilon = \frac{\beta}{4tan\theta} \quad \text{(S6)}$$

The (003) peak positions (2θ) and the FWHM of the corresponding *in-situ* XRD curves were collected by software Origin 8.5. Then, the corresponding particle size (D, nm), dislocation density δ and micro strain ε can be calculated [S3-S5].

Supplementary Figures and Tables

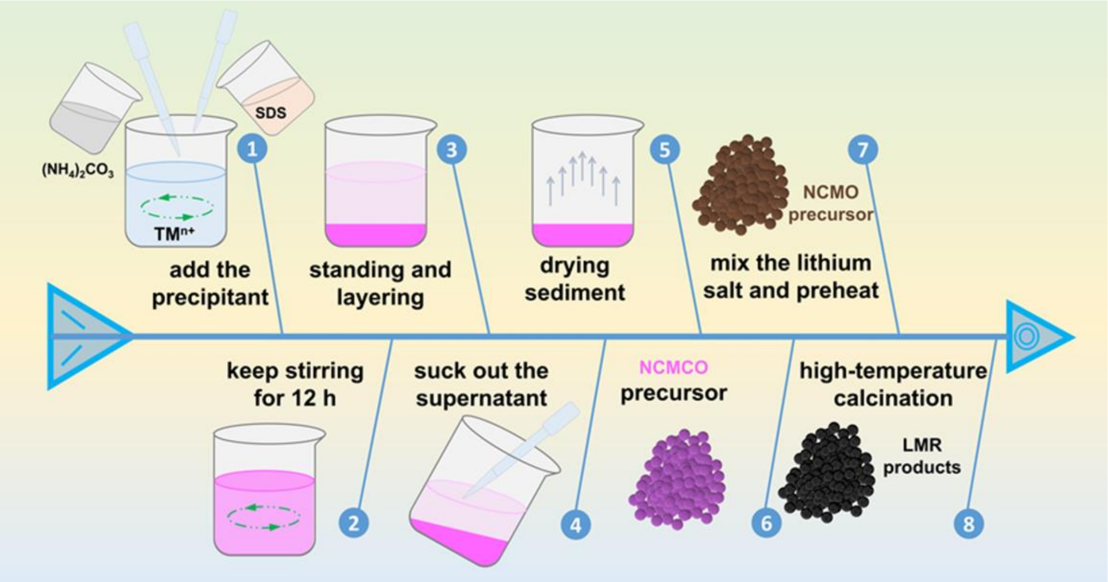

Fig. S1 Schematic depiction of the synthetic process for D-LMR sample

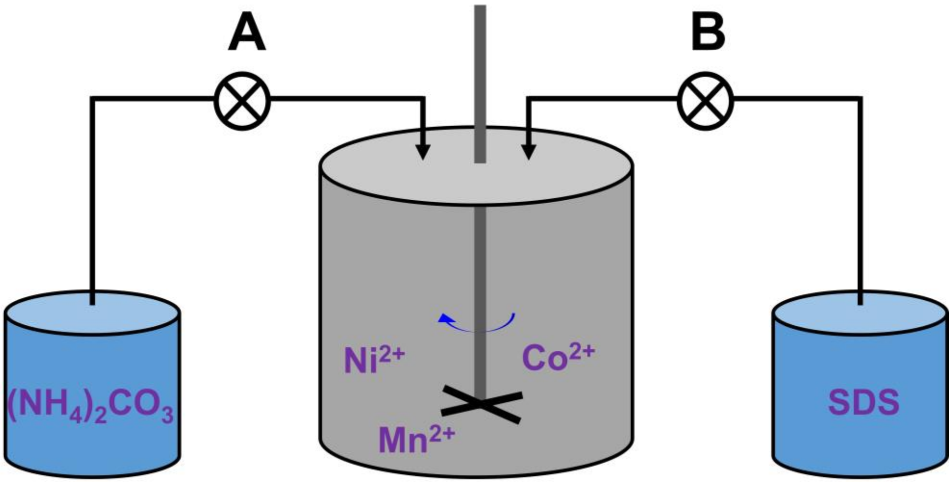

Fig. S2 Schematic diagram of the adding sequence of precipitant and surfactant

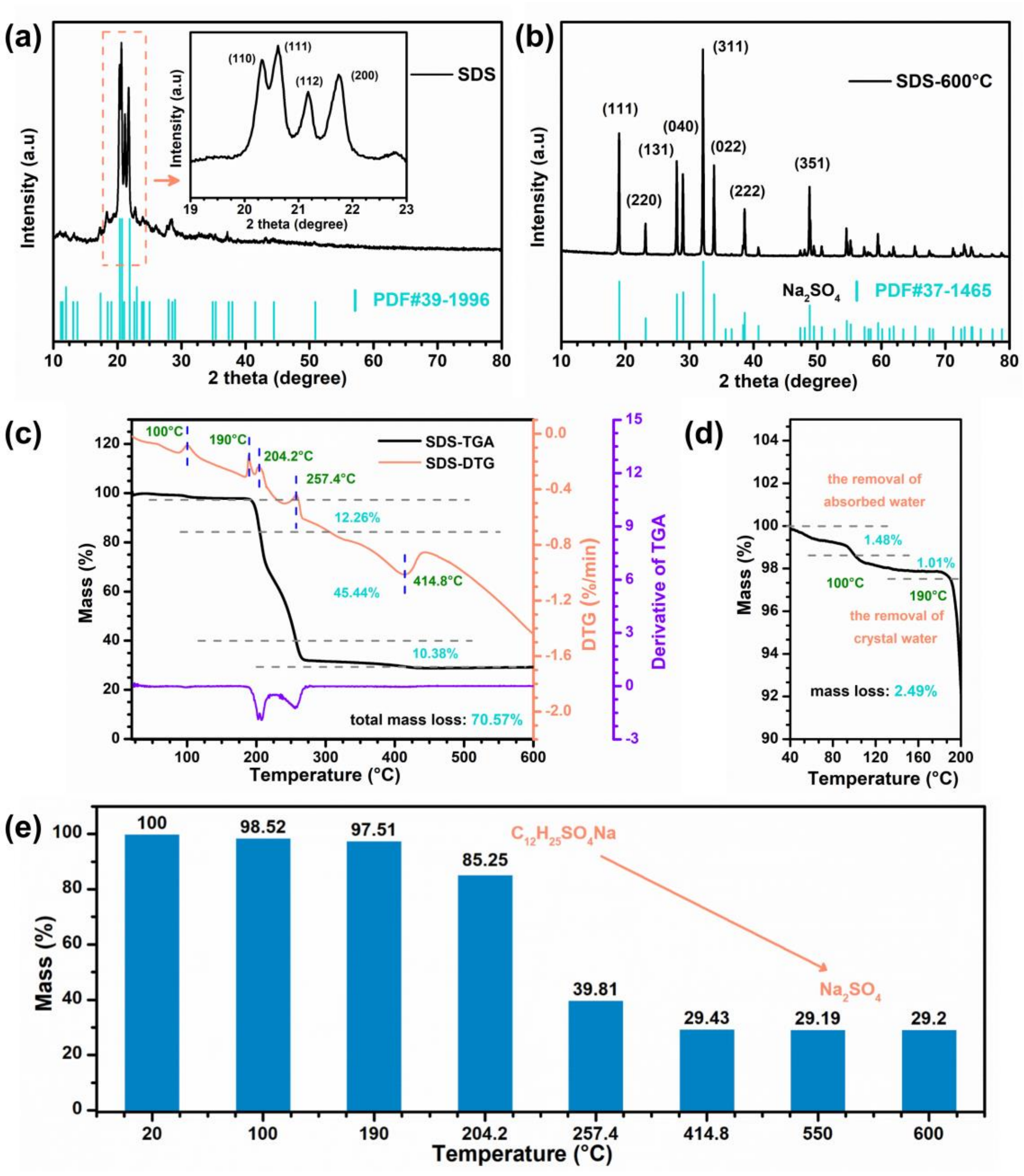

Fig. S3 XRD patterns of (**a**) pure SDS powders and (**b**) the final product of SDS after calcination at 600 °C for 5 h. (**c**, **d**) TGA and DTG curves of SDS powder. (**e**) Mass changes of SDS sample as the temperature increases

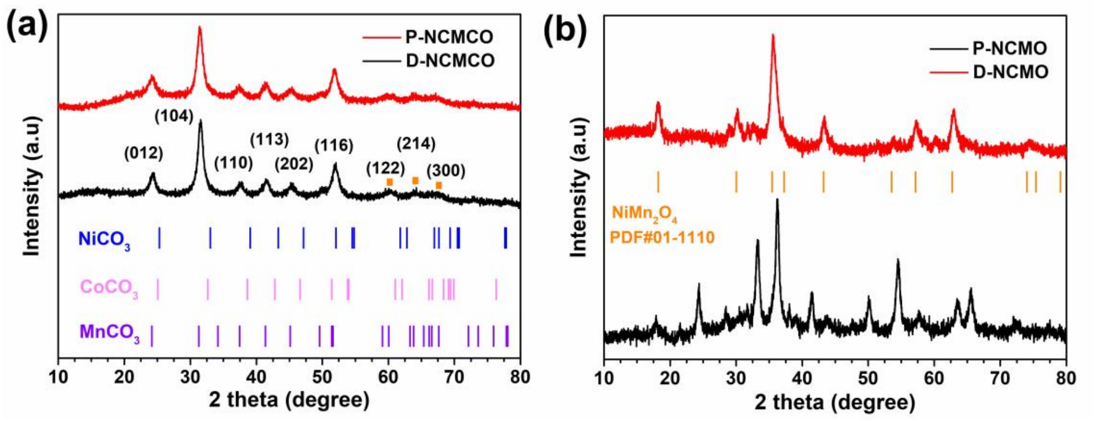

Fig. S4 Powder XRD patterns of carbonate precursors (**a**) and corresponding oxide precursors (**b**)

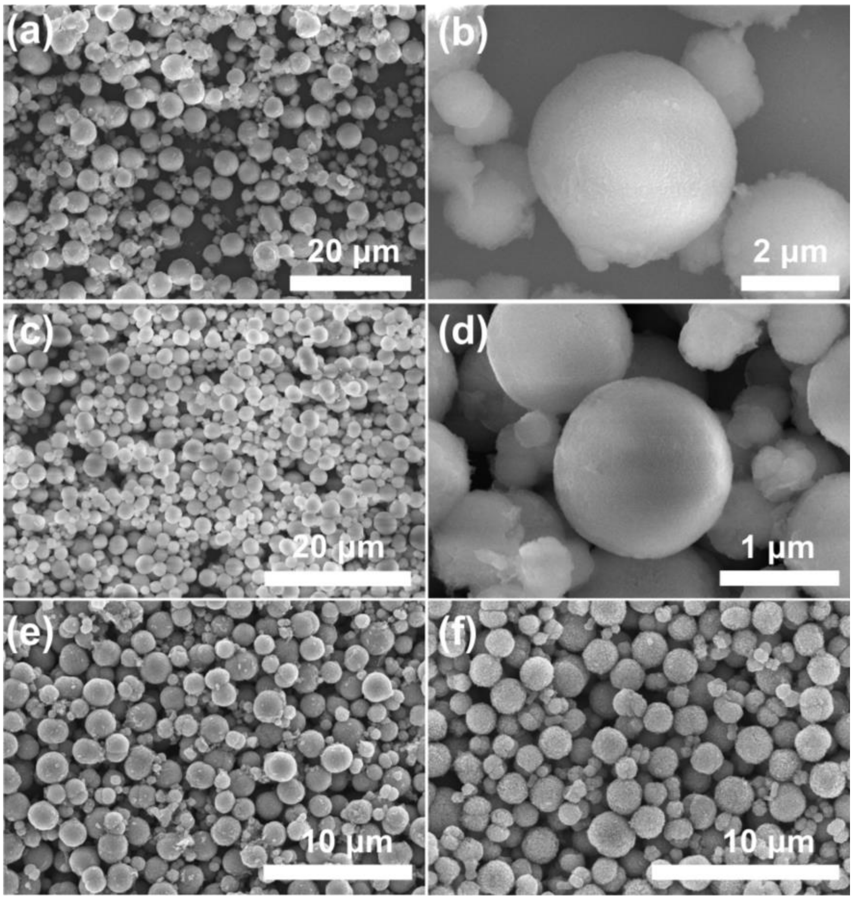

Fig. S5 SEM images of carbonate precursors for (**a-b**) P-NCMCO and (**c-d**) D-NCMCO. SEM images of oxide precursors for (**e**) P-NCMO and (**f**) D-NCMO

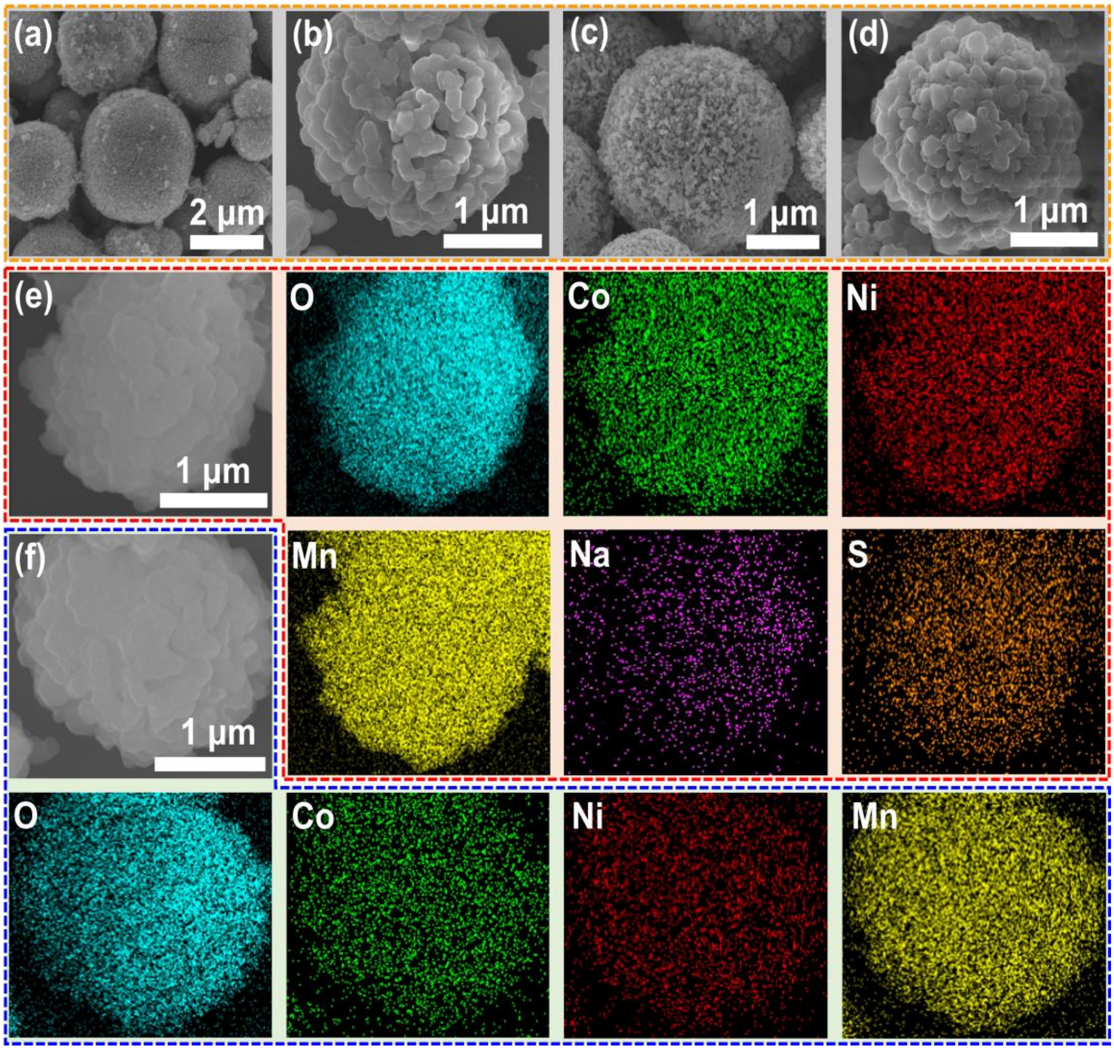

Fig. S6 SEM images of (**a**) P-NCMO and (**c**) D-NCMO. SEM images of (**b**) P-LMR and (**d**) D-LMR. The element mappings of (**e**) P-LMR and (**f**) D-LMR samples

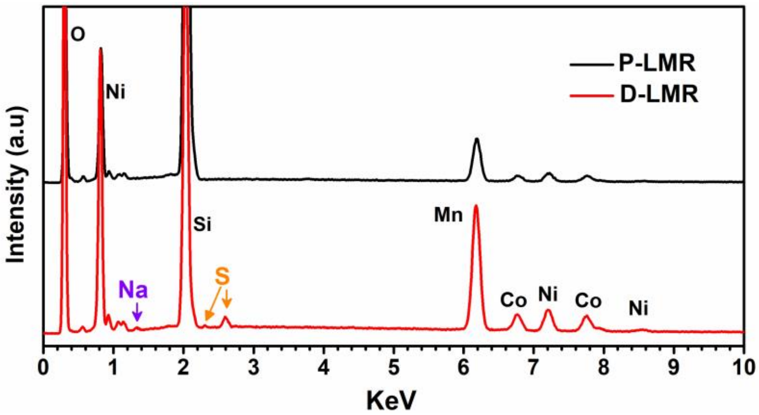

Fig. S7 EDS curves of P-LMR and D-LMR samples

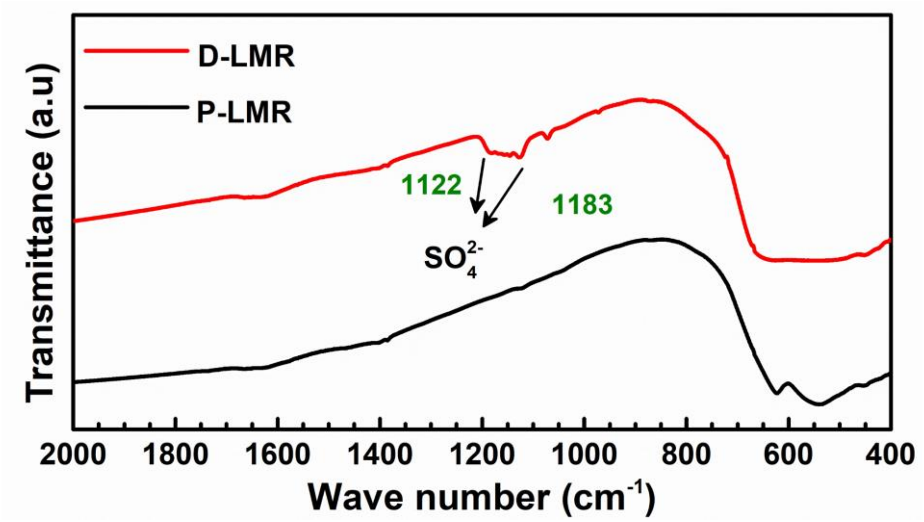

Fig. S8 FTIR spectra of P-LMR and D-LMR samples

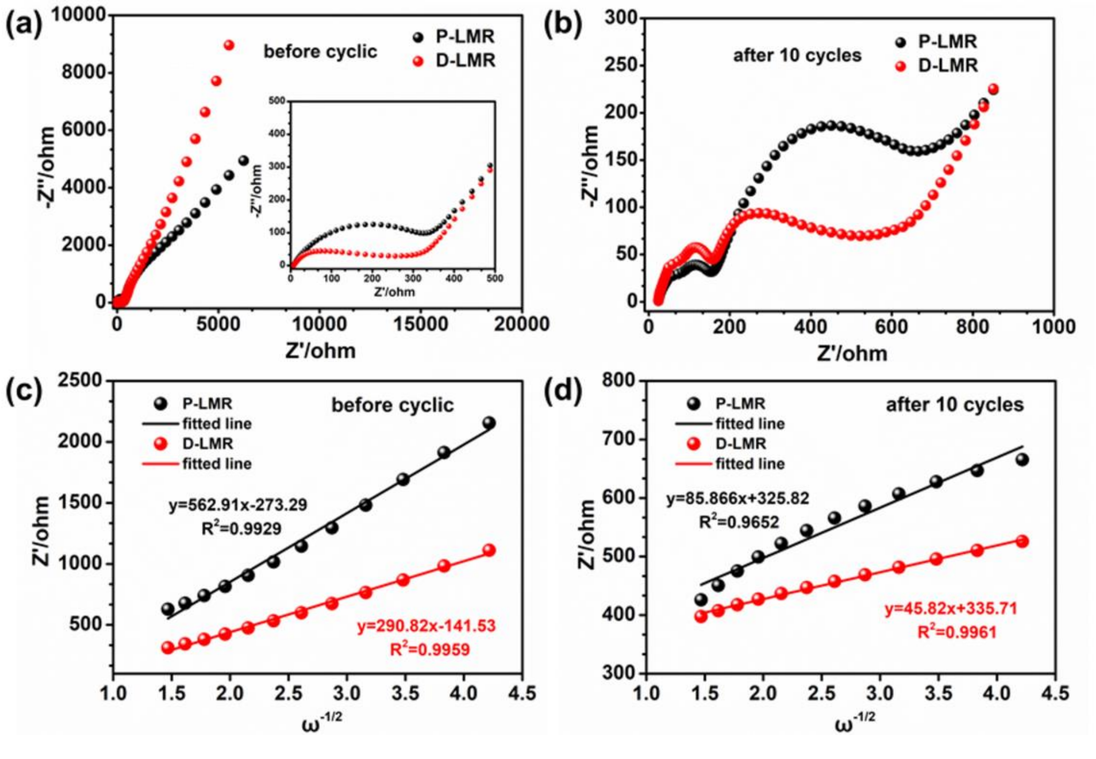

Fig. S9 Nyquist Plots of P-LMR and D-LMR cathode materials (**a**) before cycling (the inset is the enlarged view of the high-frequency region) and (**b**) after 10 cycles at 2 C rate. (**c, d**) The relationships between Z' and $\omega^{-1/2}$ for P-LMR and D-LMR samples before and after 10 cycles at 2 C

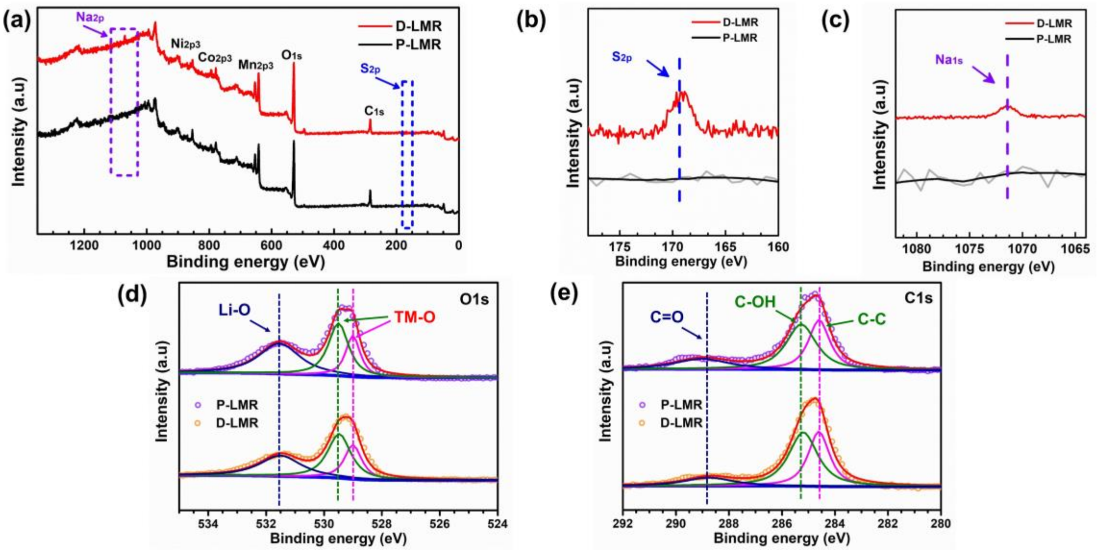

Fig. S10 XPS spectra of P-LMR and D-LMR cathode materials, (**a**) full-spectra, high-resolution XPS spectra of (**b**) S 2p, (**c**) Na 1s, (**d**) O 1s, (**e**) C 1s

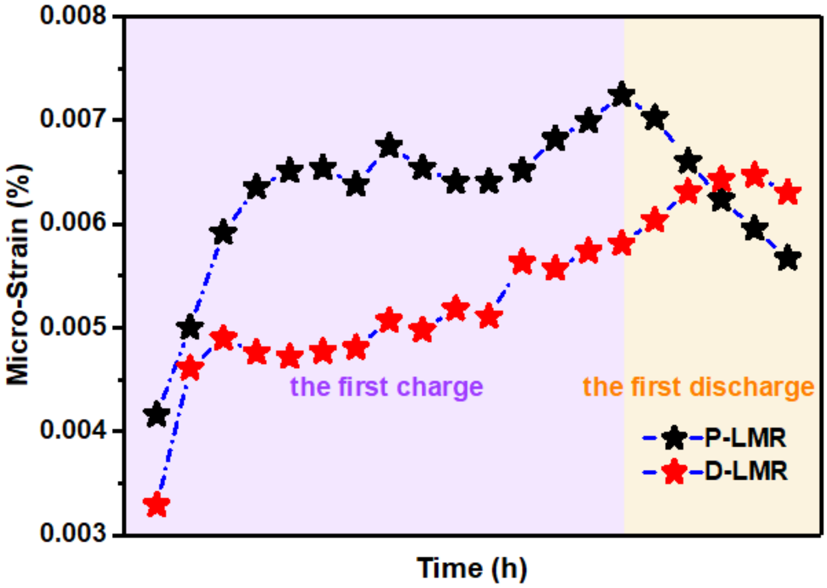

Fig. S11 Corresponding micro-strain images of the P-LMR and D-LMR samples during first charge and discharge processes

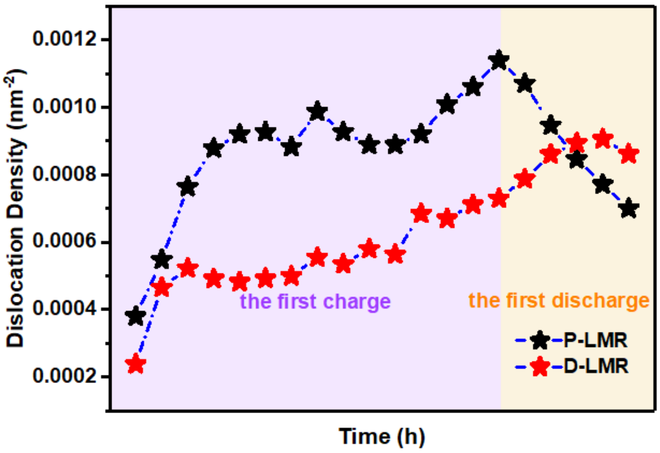

Fig. S12 Corresponding dislocation density of P-LMR and D-LMR samples during the first cycle calculating from the *in-situ* XRD test results

Table S1 Rietveld refnement results of the P-LMR and D-LMR samples

Sample	*a* (Å)	*c* (Å)	R*p* (%)	Volume
P-LMR	2.849(1)	14.227(3)	1.59	100.011
D-LMR	2.848(6)	14.237(8)	1.66	100.052

Table S2 Summary of element contents in D-LMR and P-LMR samples

Sample / Element	P-LMR Atomic percentage (%)	D-LMR Atomic percentage (%)
Mn	65.04	65.17
Co	20.14	18.18
Ni	14.82	13.89
Na	--	0.80
S	--	1.96
Total (%)	100	100

Table S3 Summary of Li^+ ion diffusion coefficients (D_{Li^+}) and the fitted impedance results for P-LMR and D-LMR electrodes before and after 10 cycles at 2 C rate (unit of D_{Li^+} is $cm^2\ s^{-1}$)

Sample		P-LMR	D-LMR
D_{Li^+} [$cm^2\ s^{-1}$]	before cycling	4.639×10^{-17}	1.994×10^{-15}
	after 10 cycles	1.738×10^{-16}	7.002×10^{-15}
R_{ct} [ohm]	before cycling	9.165	20.82
	after 10 cycles	27.6	20.21
R_{sf} [ohm]	before cycling	161.3	53.69
	after 10 cycles	324.4	207.2

Table S4 Summary of the calculated values of 2θ

Option	Data	P-LMR	D-LMR
2θ	1	18.6996	18.71244
	2	18.67579	18.67001
	3	18.64703	18.6241
	4	18.61366	18.58333
	5	18.58734	18.55914
	6	18.57514	18.55105
	7	18.57446	18.54752
	8	18.5711	18.54482
	9	18.57156	18.54172
	10	18.57446	18.54137
	11	18.57286	18.54182
	12	18.5795	18.54442
	13	18.58213	18.54557
	14	18.59064	18.54905
	15	18.58808	18.54723
	16	18.5905	18.54945
	17	18.59208	18.54949
	18	18.60936	18.55871
	19	18.61925	18.56606
	20	18.62886	18.57922

Table S5 Summary of the calculated values of FWHM

Option	Data	P-LMR	D-LMR
**FWHM**	**1**	**0.15696**	**0.12428**
	**2**	**0.18843**	**0.17364**
	**3**	**0.22249**	**0.18401**
	**4**	**0.23863**	**0.17843**
	**5**	**0.24418**	**0.1767**
	**6**	**0.24507**	**0.17855**
	**7**	**0.23919**	**0.17985**
	**8**	**0.25291**	**0.18963**
	**9**	**0.2451**	**0.18612**
	**10**	**0.23999**	**0.19381**
	**11**	**0.24006**	**0.19107**
	**12**	**0.24427**	**0.21064**
	**13**	**0.25569**	**0.20835**
	**14**	**0.26222**	**0.2147**
	**15**	**0.27167**	**0.21736**
	**16**	**0.26343**	**0.22591**
	**17**	**0.24765**	**0.23613**
	**18**	**0.2342**	**0.24066**
	**19**	**0.22365**	**0.24228**
	**20**	**0.21304**	**0.23625**

Supplementary References

[S1] W. He, H. Zheng, X. Ju, S. Li, Y. Ma et al., Multistage $Li_{1.2}Ni_{0.2}Mn_{0.6}O_2$ micro-architecture towards high-performance cathode materials for lithium-ion batteries. ChemElectroChem **4**(12), 3250-3256 (2017). https://doi.org/10.1002/celc.201700727

[S2] W. He, P. Liu, Y. Zhang, J. Lin, B. Qu et al., Utilizing the different distribution habit of La and Zr in Li-rich Mn-based cathode to achieve fast lithium-ion diffusion kinetics. J Power Sources **499**(in press), 229915 (2021). https://doi.org/10.1016/j.jpowsour.2021.229915

[S3] M. Si, D. Wang, R. Zhao, D. Pan, C. Zhang et al., Local electric-field-driven fast Li diffusion kinetics at the piezoelectric $LiTaO_3$ modified Li-rich cathode-electrolyte interphase. Adv. Sci. **7**(3), 1902538 (2020). https://doi.org/10.1002/advs.201902538

[S4] E.M.C. Jones, M.N. Silberstein, S.R. White, N.R. Sottos, *In-situ* measurements of strains in composite battery electrodes during electrochemical cycling. Exp. Mech. **54**, 971-985 (2014). https://link.springer.com/article/10.1007/s11340-014-9873-3

[S5] T. Wu, X. Liu, X. Zhang, Y. Lu, B. Wang et al., Full concentration gradient-tailored Li-rich layered oxides for high-energy lithium-ion batteries. Adv. Mater. **33**(2), e2001358 (2021). https://doi.org/10.1002/adma.202001358
